# Development of the DAGIS intervention study: a preschool-based family-involving study promoting preschoolers’ energy balance-related behaviours and self-regulation skills

**DOI:** 10.1186/s12889-019-7864-0

**Published:** 2019-12-12

**Authors:** Carola Ray, Riikka Kaukonen, Elviira Lehto, Henna Vepsäläinen, Nina Sajaniemi, Maijaliisa Erkkola, Eva Roos

**Affiliations:** 1 Folkhälsan Research Center, Topeliuksenkatu 20, 00250 Helsinki, Finland; 2 Department of Food and Nutrition, University of Helsinki, P.O. Box 66, 00014 Helsinki, Finland; 3 Department of Teacher Education, University of Helsinki, P.O. Box 9, 00014 Helsinki, Finland; 4 Philosophical Faculty, School of Applied Educational Science and Teacher Education, University of Eastern Finland, P.O. Box 111, 80101 Joensuu, Finland; 5 Department of Public Health, Clinicum, University of Helsinki, P.O. Box 63, 00014 Helsinki, Finland

**Keywords:** Intervention development, Intervention mapping, Energy balance-related behaviours, Physical activity, Food consumption, Screen time, Self-regulation skills, Preschoolers, Socio-economic differences in health behaviours, Preschool-based family-involving intervention

## Abstract

**Background:**

Preschoolers’ energy balance-related behaviours (EBRBs) and self-regulation skills are important for their later health. Few preschool-based interventions aiming to promote preschoolers’ EBRBs and self-regulation skills, simultaneously reducing differences in EBRBs, due to children’s socio-economic status (SES) background, have been conducted. This study will present the Increased Health and Wellbeing in Preschools (DAGIS) intervention development process applying the Intervention Mapping (IM) framework.

**Methods:**

The development of the DAGIS intervention study, a preschool level clustered randomized controlled trial (RCT), was based on the IM framework. The protocol in IM guides the development process of an intervention through six steps: needs assessment and logic model of the problem, programme outcomes and objectives, design of the programme, production, implementation plan, and evaluation plan.

**Results:**

The needs assessment, part of the step 1 in IM, yielded the base for the DAGIS logic model of change. The model includes objectives related to changes in children’s EBRBs, self-regulation skills, and in psychosocial and physical environment that is determined by parents and early educators. A 22-week programme was developed, and materials for preschools and families were produced. A feasibility study of the recruitment processes, acceptability of the materials and methods, and implementation was conducted. The DAGIS intervention study was conducted September 2017–May 2018 as a clustered RCT including a comprehensive effectiveness and process evaluation. The process evaluation was run throughout the intervention targeting preschools and families.

**Conclusion:**

A preschool-based family-involving programme was developed in the DAGIS intervention study by applying the IM protocol. It was a time- and resource-consuming process. However, the systematic planning, development, and running of the programme have reinforced a comprehensive evaluation, which is a strength in the intervention. The results from the evaluation will enhance the knowledge of how to promote EBRBs and self-regulation skills among preschoolers, and diminish SES differences in them.

**Trial registration:**

ISRCTN57165350 (Prospectively registered January the 8th, 2015).

## Background

Children’s physical activity (PA), sedentary behaviour, and food consumption are commonly called energy balance-related behaviours (EBRBs). EBRBs and stress regulation are important for children’s current and future health and wellbeing [1–3]. Previous studies show that a socio-economic status (SES) gradient exists at least in some of children’s EBRBs. Children from low SES backgrounds have more screen time [4], less PA [5, 6], and lower intake of fruit and vegetables [4, 7]. Some studies show an association between children’s low SES backgrounds and higher stress levels [8].

In order to promote children’s further health and wellbeing it is important to focus on promoting children’s healthy EBRBs, as well as lower the effects of stress. It has been shown that self-regulation skills are highly linked to stress levels, and therefore strengthening children’s self-regulation skills should be given high priority when aiming to diminish stress levels in children [9, 10]. Self-regulation as a concept is multidimensional, and it has been described as a construct including both emotional and behavioral self-regulation [10]. Shortly described, emotional self-regulation is the capacity to be able to recognize own feelings and stay calm in stressful situations, whereas behavioral self-regulation relates to impulsivity, or inhibitory control [9, 10]. Poor self-regulation skills have been associated with less favorable EBRBs, such as higher intake of palatable food, or using food as a reward, and further with higher BMI later in life [11, 12]. Recently, it has been proposed that strengthening children’s self-regulation skills, along with the promotion of healthy EBRBs, leads to more efficient results in children’s EBRBs, as well as a healthy weight, than by only promotion healthy EBRBs [9, 13]. The approach to promote children’s healthy EBRBs and simultaneously strengthening children’s self-regulation skills, has been applied at least in one intervention study [10]. In general, the effects on children’s EBRBs were not significant in the study, although the sugary drink consumption decreased more among those belonging to the intervention arm that simultaneously promoted healthy EBRBs, and strengthened self-regulation skills, compared to the other intervention arms in which both components were not included [10]. One explanation to the poor effects could be that families were not engaged enough in the preschool intervention. Family engagement in preschool interventions has been emphasized in reviews as a crucial part for successful interventions in children [14]. Another prominent aspect when conducting interventions including families is the SES gradient, which exists in children’s EBRBs [4–7]. In the intervention planning phase, the researchers should try to plan activities, which are able to reach those who need it most, in this case families with low SES backgrounds, and further on reduce the SES differences in EBRBs. In order to avoid stigmatization based on SES in a population wide intervention, an appropriate way might be to apply the proportionate universalism approach [15]. In this approach, the intervention is delivered to the whole target population in a similar manner, and the intensity of the intervention is adjusted according to the needs of children from low SES backgrounds [15]. Few intervention studies have focused on narrowing SES differences in young children’s EBRBs [16, 17].

In Finland, early childhood education and care centres, hereafter preschools, are good arenas to promote children’s EBRBs and strengthen self-regulations skills. Most preschools are municipality-driven and open for all children. About 86% of all 5-year-olds attend preschool in Finland, the percentages being slightly lower among 4- and 3-years old, about 83 and 78% respectively [18]. Children usually attend a preschool near their home independently of their SES background, which means that most preschools have children with diverse SES backgrounds. Therefore preschools have huge possibilities to act as health-promoting arenas, and narrowing possible SES differences in children’s EBRBs and stress regulation. The current National Core Curriculum for Early Childhood Education and Care 2016 emphasizes that preschools, in collaboration with families, should promote children’s health behaviours and wellbeing [19]. The food recommendations for early childhood education and care, as well as the recommendations for physical activity in early childhood, are emphasizing the preschools’ role in promoting EBRBs [20, 21].

Even though an arena for reaching many children with mixed SES background exists, the intervention planning still needs to be carefully conducted. Kok et al. [22] have proposed that effective interventions need to fulfil three conditions. Firstly, the intervention needs to target a determinant that is predicting the behaviour that the intervention aims to change. Secondly, the intervention should be able to change the determinant. Thirdly, the intervention needs to be conducted in a way that parameters are preserved in the practical application. The practical application should be accepted by the target population, considering the culture and the context in which it is conducted. In the Medical Research Council (MRC) guide for developing complex interventions, it emerges that there is a need for deeper work in the early stages of intervention planning [23]. The MRC guide emphasizes that there is a need to take the local context into account in the development of the intervention and piloting is an important phase in order to develop a successful complex intervention. A complex intervention is a programme that includes several interacting components, a high number of behaviours, and targets several levels of organizations [23]. Durlak and DuPre [24] concluded in their meta-analysis that the context and readiness of the implementers are relevant for successful implementation of interventions, and should be measured. Still, process evaluations have often been small-scale alongside effect evaluations, and knowledge of the crucial factors for a high-degree implementation of an intervention in the preschool and family setting is scarce [24]. A useful tool for developing complex interventions is the Intervention Mapping (IM) protocol, which enables a systematic way of designing, implementing and evaluating an intervention [22]. The planning process should be based on theoretical, empirical and practical information. The IM protocol is comprised of six steps: 1) needs assessment; 2) forming change objectives and model of change; 3) designing a programme; 4) producing materials; 5) planning, adoption, and implementation; and, 6) evaluation of the intervention [25]. The IM protocol states that the first two steps are of utmost importance.

The Increased Health and Wellbeing in Preschools project (hereafter called the DAGIS project) emerged from the knowledge of SES differences in preschoolers’ with overweight and obesity in Finland [26]. A long-term goal of the DAGIS project is to diminish SES differences in weight status among preschoolers. The main aim for the DAGIS intervention study, here presented, is to promote preschoolers’ EBRBs and strengthen self-regulation skills simultaneously reducing SES differences in them. A useful approach for an intervention, which includes all children independently of SES background, and simultaneously reduces SES differences, is the proportionate universalism approach [15]. Briefly, those with the highest needs should benefit most from the intervention [15]. The objectives of this paper are to present the planning process of the DAGIS intervention study, and to describe how the IM protocol has been applied throughout the DAGIS project.

## Methods

In this paper, the methods section will shortly describe the six steps in the IM protocol [25], and briefly, which are the corresponding steps in the DAGIS intervention study. The results section describes the main results yielded in each step of the DAGIS intervention development. It also presents how the results were used in moving forward on the IM protocol steps.

### Steps 1 and 2: understanding the problem and determining the theory and evidence base

The aims of the first and second steps in IM are to better understand the problem at hand, to identify key behaviours and their determinants related to the problem, and to specify the objectives for change [25]. In the DAGIS study, a comprehensive needs assessment with two central aims was conducted:

1. Gain insight into environmental barriers and facilitators related to preschool children’s EBRBs and stress regulation specifically in the Finnish context
2. Explore EBRBs and stress regulation and their determinants in which SES differences exist among Finnish preschoolers.

A DAGIS socio-ecological model for children’s EBRBs was developed in early stages of the project [27]. The model was used as a guiding tool when planning the comprehensive needs assessment. The needs assessment in the DAGIS study included three steps: 1) focus group interviews, 2) an informal literature review and 3) a cross-sectional survey. The steps all examined preschool children’s PA, sedentary behaviour, screen time, dietary behaviour, stress regulation and factors related to EBRBs. A steering group for DAGIS was established. The group members were stakeholders with different backgrounds, including directors of early education, preschool managers, early educators, researchers, and representatives of Finnish parents’ league (representing parents), children’s health-promoting organizations, and health promoters in the field of young children. The steering committee met up about twice a year. Proceedings and *obstacles in the DAGIS study were discussed, and* knowledge and experiences were shared.

The focus group interviews in autumn 2014 were carried out in areas where, according to municipality statistics, a slightly higher proportion of the inhabitants had lower SES backgrounds. The interviews were conducted separately with parents of preschool-aged children (6 groups, total *n* = 17) and early educators (4 groups, total n = 17). The focus groups shed light on early educators’ and parents’ thoughts on their own roles in influencing children’s EBRBs, as well as important barriers and facilitators for these behaviours. The focus group interviews have been presented in more detail in two published articles [28, 29]. The informal literature review updated the research group about children’s EBRBs, determinants for children’s EBRBs, and the EBRBs where SES differences exist. It also provided knowledge on previous interventions carried out in preschool settings with similar aims. The cross-sectional survey was conducted between autumn 2015 and spring 2016 in eight municipalities in southern and western Finland, with 66 preschools, and 864 participants. The survey comprehensively elaborated the current state of PA (measured by accelerometers for 7 consecutive days), sedentary behaviour (accelerometers), screen time (7-day screen time diary recorded by parents), dietary intake (food frequency questionnaire and 3-day food records), children’s temperament (Children’s Behavior Questionnaire- the very short form) [30], children’s stress levels (long-term measured via hair cortisol concentration, short-term stress measured via salivary cortisol-, and alpha-amylase), and their determinants among Finnish preschoolers [31]. In addition, the survey provided data on socio-economic differences in children’s EBRBs, and explored psychosocial and physical environmental determinants for EBRBs. Mediation analyses were planned to be conducted in order to explore which determinants are of importance for the associations between low family SES and children’s EBRBs. The analyse plans for the survey data also included moderation analysis to explore whether family SES moderated the associations between determinants for children’s EBRBs and children’s EBRBs. The examined themes for the survey were derived from the DAGIS socio-ecological model [27], the focus groups, and the existing literature.

Objectives for children’s and parents’ behaviour changes were identified, and a logic model for change was developed based on the findings. Literature was reviewed with a focus on behaviour-change theories that have shown to be useful and effective in interventions conducted in preschool settings. The logic model of change was complemented with constructs that were identified as essential according to the selected behavioural theories.

### Steps 3 and 4: designing the DAGIS programme and producing programme materials

In the third step of the IM protocol, the aim is to design and create the actual concrete programme: its themes, components, and general structure [32]. Furthermore, the third step includes decisions about which behaviour-change methods are used and what kinds of practical applications they are turned into. In the fourth step of the IM protocol, the aim is to refine the programme design and components, if needed, and produce the programme materials.

In accordance with the third step, the contents, components, and structure of the DAGIS programme were designed during autumn 2016 and spring 2017. The process included four main methods: 1) workshops for parents and early educators, 2) workshops for the steering group, 3) an informal literature review, and 4) feasibility testing of the chosen strategies and materials.

The parents’ and early educators’ workshops were conducted in autumn 2016. Altogether eight workshops were held, of which two were only for early educators, two only for parents, and four both for parents and early educators together. The aim of the workshops was to gather participants’ ideas about what could be done in real life to promote healthy habits at home and at preschool, and what kind of delivery methods and materials would be suitable and acceptable to them. During a steering group meeting, group members attended workshops in which they could generate ideas about how to successfully recruit families to the intervention study, and share thoughts about appealing materials and methods for the intervention. The aim of the informal literature review was to identify effective behaviour-change methods and suitable practical applications to be adapted to the DAGIS programme. The review included searching through the international scientific literature as well as mapping out materials produced by other national health-promoting organizations. Some materials were developed specifically for the DAGIS programme because suitable materials for influencing all targeted determinants did not exist. The feasibility tests of developed materials and methods were conducted in June 2017 in two preschools. Parents (*n* = 19) provided feedback regarding developed materials and methods through questionnaires. Early educators (*n* = 4) gave their feedback by informal interviews. Simultaneously, recruitment procedures for the study, and feasibility of the evaluation questionnaires were tested in five additional preschools. Feedback related to the planned family recruitment process was received through an early educator’s informal interview in one of the preschools. Feasibility of the planned evaluation questionnaires was assessed based on the questionnaire responses received (parents’ questionnaires *n* = 16; early educators’ questionnaires *n* = 11).

### Step 5: planning adoption and implementation

The purpose of the fifth step in the IM protocol is to plan: 1) how people who adopt and implement the programme can be motivated to take it up and engage in the activities; 2) materials and programme objectives; and, 3) how to maintain the programme [25].

Adoption and implementation of the DAGIS intervention were planned simultaneously with the intervention design and materials. The literature was analysed with a focus on early educators’ motivation and training because they were recognized key people for the implementation of the DAGIS intervention. The DAGIS steering group included experts from the field of early childhood education who added valuable knowledge about adoption and implementation of interventions in preschool settings. The feasibility testing in step 4 added information on adoption and implementation of the programme.

### Step 6: evaluation

In the last step of the IM protocol, the evaluation should be planned [25]. Programme effectiveness and the process should be evaluated [25]. Evaluation of the DAGIS intervention was planned in tandem with the development of the programme, and refined and finalized as the programme was running. The evaluation of the effectiveness was planned based on the logic model of change formed in step 2. The DAGIS intervention study was conducted as a preschool level clustered randomized controlled trial. Power calculations based on results from the DAGIS cross-sectional survey were made to obtain the needed sample size to detect changes in children’s EBRBs. The RE-AIM (reach, effectiveness, adoption, implementation and maintenance) framework was a crucial guiding tool in planning the evaluation [33]. The DAGIS intervention also applied several other frameworks and guidances in designing the process evaluation [23, 34–36]. The process evaluation focused on the activities and inputs in the intervention, and the implementation process. The DAGIS intervention study was approved as ethically acceptable by the University of Helsinki Ethical Review Board in Humanities and Social and Behavioral Sciences in May 2017 (22/2017).

## Results

This section describes results gained in each step of the planning process and how the results have been applied in the DAGIS intervention development.

### Results in steps 1 and 2: understanding the problem and determining the theory and evidence base for the DAGIS intervention

#### Factors influencing preschool children’s health behaviours and barriers to change

The focus groups yielded valuable knowledge about the home and preschool context as promoters or barriers for EBRBs and results have been reported in detail elsewhere [28, 29]. Parents talked about themselves as role models for both PA and intake of sugary foods and drinks. Parents also described that their children consumed many sugary foods daily. Still, when asking them, they were not concerned about the fairly high sugar intake. Parents did not either see a problem in their children not being physically active enough. Early educators recognized that excessive sitting time existed at preschool. Still, many early educators felt that it was an important task to teach children to sit because children need this skill in school. Early educators acknowledged themselves as role models for PA, especially when outdoors. However, many mentioned that they do not have the time to act as role models, or they are not motivated to encourage PA, as children were physically active without any encouragement [28]. Focus group results were applied when designing the questionnaires for the comprehensive survey and the intervention measurements. Further, the results were utilized for development work of the logic model of change and when designing the programme and its contents.

#### Socio-economic differences in children’s health behaviours and stress

In the cross-sectional survey, the family SES was assessed comprehensively, and the results of SES differences in children’s EBRBs have been reported in detail elsewhere [31]. Lower parental education (answering parent) was associated with children’s higher screen time (measured by a 7 day screen time diary), a more frequent consumption of sugar-sweetened beverages and sugary everyday foods e.g. flavoured yoghurts and quarks, sugar-sweetened cereals, measured by a food frequency questionnaire [31]. Measuring food intake by 3-day food records showed that children whose parent was lower educated (high school level or lower education) ate fewer vegetables (in grams) than children whose parent had a master’s degree or higher education [31]. The DAGIS survey did not show any SES differences in children’s long-term stress, which was measured by analysing hair samples from each child (cortisol concentrations).

The literature review supported the survey findings of less beneficial EBRBs among children from low SES backgrounds, and most evident SES differences were found in sugary food and beverage consumption [4, 7, 37], and in screen time [4, 38, 39]. The literature review also indicated SES differences in children’s fruit and vegetable intake [4, 7]. Furthermore, the literature review showed that a low SES background predisposed children to higher stress levels [8], and different stress measures were shown to have an adverse association to health behaviours and weight [2, 9, 40]. Self-regulation skills was proposed to play an important role in moderating the effects of stress on health behaviours [9, 40], and thus, strengthening the self-regulation skills could help to decrease SES differences in health behaviours. In addition, previous studies suggested that the effects of an intervention might be higher when including strengthening of self-regulation skills along with promoting healthy EBRBs [10, 13].

#### Mediating factors between SES and children’s EBRBs

In mediation analyses, the data from the DAGIS cross-sectional survey was used. As the found SES differences in children’s EBRBs were related to parental educational level, the mediation analyses used parental educational level as the independent variable and children’s screen time and sugary food and drink consumption as dependent variables. Testing for mediation factors yielded following mediators, which have been published elsewhere [41]: 1) parents’ views on acceptable screen time for children, 2) their own screen use in front of their child, 3) importance they place on limiting child’s screen time, and 4) feelings of societal pressures to use screens. In not so far published analysis, the associations between parental education and sugary food and drink consumption were mediated by: 1) availability of these foods at home, and 2) parents’ views on acceptable consumption frequency of these foods and drinks (not published).

Literature on mediators between SES and preschoolers’ EBRBs is scarce. One study showed that parental television viewing and having a television in the child’s bedroom mediated the associations between parental SES and children’s screen time [39]. The availability of, and the parent’s permissiveness towards consumption of sugary foods and drinks, as well as the parent’s self-efficacy to serve water instead of soft drinks were in one study identified as significant mediators for association between SES and sugary food and drink consumption [42].

#### Theoretical underpinnings of the DAGIS intervention

To influence children’s EBRBs, the behaviours of the adults around the children must be addressed. Therefore, there was also a focus on theories that were important for changing the behaviours of the adults. The Social Cognitive Theory (SCT) [43], Theory of Planned Behaviour (TPB) [44], and Self-Determination Theory (SDT) [45] were chosen as the framework for the intended behaviour changes in adults and further on in children’s EBRBs (see Additional file 1: Figure S1). The reason for choosing these theories is twofold. Firstly, many central components in the above-mentioned theories were identified in the focus group interviews that took place in 2014, and they were found to be significant mediators in the associations between parental education and children’s EBRBs in the DAGIS cross-sectional study data [41] Secondly, these behaviour change theories have been shown to be practical tools to conceptualize behaviour change, and their application has in intervention studies yielded actual behaviour change [46, 47].

#### Development of the DAGIS logic model of change

Based on the findings from the survey and the literature review, three programme objectives for changing children’s EBRB and self-regulation skills were defined (see the secondary outcomes in Table 1). Objectives were formulated so that the intervention did not only pursue to decrease unwanted EBRBs by reducing screen time, and sugary foods and drinks consumption, but also to influence beneficial EBRBs by increasing PA, and fruit and vegetable consumption. By promoting beneficial EBRBs, families were approached also with a positive encouraging message. The intention to change is more likely when you think that advantages outweigh disadvantages [44]. The last objective to strengthen children’s self-regulation skills was formed based on the literature findings that linked children’s weight and EBRBs with their self-regulation skills [2, 9, 40].

**Table 1:** The DAGIS logic model of change

Inputs	Activities	Primary outcomes, in adults 2017–2018		Secondary outcomes, in children 2017–2018	Long-term outcomes (for children)
		Short term	Intermediate		
Research personel	Preschools	Changes: Norms about screen time & sugary everyday foods and drinks	Changes: Role modelling	Excessive screen time diminishes & Physical activity (PA) increases	Prevalence of overweight decreases
Funding	Group component for parents, preschool personel & children	Knowledge,	Availability and accessibility	Sugary foods and drinks consumption diminishes & Fruit and vegetable consumption increases, changes to less sugary products	Wellbeing and learning abilities increases
		Attitudes		Self-regulation skills strengthens	
Materials	Home component	Motivation, engagement			
Collaborators		Awareness			
		Skills and self-efficacy			
		Social support			

Further, the most important mediators between family SES and children’s EBRBs in the DAGIS survey were parents as role models, and the availability and accessibility of screens and sugary foods ( [38] and unpublished results). Guided by the theoretical underpinnings and findings from the needs assessment, we further defined determinants that we needed to influence in order to change the behaviour of children (see the primary outcomes in Table 1). Based on these results and the theoretical underpinnings, the DAGIS logic model of change was developed (Table 1). Children’s EBRBs, which are the behaviours the DAGIS intervention aims to change, form the secondary outcomes. The primary outcomes are behaviours or activities in adults that need to be changed in order to have a change in children’s EBRBs and self-regulation skills. The primary outcomes include intermediate and short-term outcomes. The intermediate outcomes are those determinants that in the survey, independently of the behavioural outcome, were mediators in the associations between parental SES and children’s EBRBs. The short-term outcomes were determinants derived from both the mediator analyses of the DAGIS survey, and from behavioural theories.

### Results in steps 3 and 4: designing the DAGIS intervention programme and producing programme materials

#### Programme themes, components, and scope

Programme themes founded were supporting children’s self-regulation skills, increasing PA and intake of fruits and vegetables, and decreasing screen time and intake of sugary foods and drinks. Most evident socio-economic differences were in the needs assessment found in EBRBs that do not occur at preschools, but in the home environment, or are factors related to parenting practices. This fact resulted in a decision to develop a component with a substantial amount of materials for parents. The decision was supported by the literature that suggests that a high level of parental involvement is an essential part of effective interventions in changing children’s EBRBs [47, 48]. Further, we wanted to have an intervention component implemented at preschool. In Finland, supporting self-regulation is one of the main goals in national curriculum for early childhood education and care [19]. Therefore, the component at preschools emphasized the promotion of self-regulation skills. Implementing a preschool component reassured that intervention themes, both self-regulation and EBRBs, will be discussed with children irrespective of their parents’ interests. With a preschool component, we were also able to strengthen children’s active participation, which was emphasized in the parents’ and early educators’ workshop discussions. They felt that parents who are harder to reach could be motivated, if the messages come from their own child, in addition to other materials aimed directly for the parents.

#### Choosing behaviour-change methods and practical applications

Once we identified our objectives for children’s and adults’ behaviour changes, we used lists provided by Bartholomew et al. [25] and Michie et al. [49] to define change targets and to find theory-based methods to guide behaviour changes (Table 2). Several behaviour-change methods were identified; among them were active learning, direct experience, providing information on behaviour-health links, mobilizing social networks, and providing social support. We used literature to guide the selection of behaviour-change methods. Literature findings suggested that effective interventions more commonly used techniques prompting specific goal setting, environmental restructuring [50], providing general information on behaviour-health links, and social support [51, 52].

**Table 2:** Practical strategies, methods and behaviour-change targets of the DAGIS intervention programme in accordance with the IM protocol^a^

Intervention level	Practical strategies	Behaviour-change methods	Change target
Preschool	Early educators’ trainings	Information about social and environmental consequences^b^	Attitude
		Framing^b^	Attitude
		Information on how to perform the behaviour^b^	Skills, capability, self-efficacy
		Verbal persuasion about capability^b^	Skills, capability, self-efficacy
		Mobilizing social networks^a^	Social influence
	Manuals	Information on how to perform the behaviour^b^	Skills, capability, self-efficacy
	Meeting with preschool managers	Discussion^a^	Motivation Knowledge
	Mentoring visits	Discussion^a^ Social support (practical)^b^	Knowledge Motivation Capability
	Booster e-mails with pedagogical tips	Information on how to perform the behaviour^b^	Motivation Skills, capability
Preschool & family	Activity afternoon	Active learning^a^	Attitude
		Mobilizing social support	Social influence
Family	Educational letters & DAGIS e-mails	Persuasive communication^a^	Attitude
		Prompting goal setting^a^	Skills, capability
		Information on how to perform the behaviour^b^	Skills, capability
		Restructuring the physical environment^b^	Skills, capability
		Identification of self as role model^b^	Awareness
		Normative information about other’s behaviour	Social influence/social norm
	Personal feedback on children’s measured EBRBs and information about the measured average of study participants’ behaviours	Feedback^a^	Awareness Motivation
		Normative information about other’s behaviour	Social influence/social norm
	Map application	Social support^3^	Social influence
Family and child	Bingo board	Active learning^a^	Skills, capability
		Direct experience^a^	Attitudes
Child	MindUp™ curriculum	Guided practice^a^	Skills, capability
		Improving physical and emotional states^a^	Skills, capability
		Active learning^a^	Skills, capability
		Using imagery^a^	Knowledge
	Activities related to EBRBs	Active learning^a^	Skills, capability
	Storybooks	Modeling^a^	Skills, capability
		Using imagery^a^	Knowledge

^a^Bartholomew et al. [25, 32]

^b^Michie et al. [49]

The behaviour-change methods were converted into practical strategies (Table 2). Information from similar interventions, results from the workshops, and existing materials produced by other organizations were used. For example, the workshop discussions emphasized the importance of sense of community, active participation of children, mutual fun activities for parents and children, and more communication between early educators and parents about skills practiced at preschools. Based on the aforementioned comments, several levels were decided to be included in the intervention (see Table 2). Also a shared preschool and family level was included, and early educators were instructed to organize activity afternoons at the preschool. At these afternoons parents and children together would conduct different theme activities. The afternoons created a possibility for parents to discuss the themes with other parents and early educators. During the afternoons, the early educators were also able to make visible for parents the themes covered and skills practiced at the preschool.

#### Designing the programme, content, and materials for preschools and families

Because we had chosen to develop a preschool-based intervention that involved families, we needed to develop a programme that had content for both preschools and families. For the preschool component, a core international programme, the MindUp™ curriculum, was chosen [53]. The MindUp™ curriculum has its foundations in theory and research on cognitive developmental neuroscience, mindfulness, and positive psychology [54]. The curriculum aims to develop self-awareness, self-management, and self-regulation. Many activities in the programme relate to physical activity and food. In Finland, MindUp™ has been applied in multiple preschools. DAGIS researchers have been involved in evaluating the implementation of the MindUp™ in preschools (not published), and through the early educators evaluation, we received valuable knowledge of best practices for adapting and implementing the curriculum. The core activity in the curriculum is the short relaxation moment, which is conducted three times daily in the preschools. The curriculum did not address all the determinants that we had identified as important and aimed to change through the DAGIS intervention. Thus, additional materials and activity ideas were developed, such as two storybooks and several manuals. Some materials were adapted from other existing materials that promote healthy EBRBs [55, 56]. The family materials included some issues about children’s self-regulation from the MindUp™ themes. However, in the family materials, we focused on issues that are relatively hard to change by preschools (e.g. children’s screen time, consumption of sugary foods and beverages) (occurred at home, unpublished results from the survey). The practical strategies and contents are presented in more detail in Table 3.

**Table 3:** Practical strategies, their contents and implementation

Intervention level	Practical strategies	Strategy contents	Implementation
Preschool	Early educators’ trainings	Intervention aims, timetable, materials and theoretical background Group discussions on intervention implementation Manuals were distributed, and their contents were scrutinized Based on MindUp™ training	Before the intervention started, all early educators participated in 5–5.5-h training; at the halfway point of the intervention, the early educators participated in an additional 2.5–3-h training
	Manuals	Short theoretical introduction to the contents of the theme Activity ideas to do with children at preschool Connection of the theme and activities to the national core curriculum for early education and care Materials that should be distributed to parents during the theme period	One manual for each theme (altogether five themes) was distributed during the training sessions
	Meeting with preschool managers	Informal meeting aiming to recognize the problems that preschools might have with programme implementation Meeting informed the researchers how each of the preschools preferred the mentoring visits to be organized	Was organized 2 weeks after early educators’ trainings; managers from all intervention preschools gathered together in the town hall where the meeting was held
	Mentoring visits	Varied slightly according to the needs/wishes of the early educators and manager: either an organized meeting with early educators and manager or free discussion only with early educators in each group about how the intervention implementation had started	Were organized 2–3 weeks after the intervention start at the preschools
	Booster e-mails with pedagogical tips	Reminder that a new theme was supposed to be started at preschools Additional practical activity ideas about how the theme could be discussed with children at preschool	Were sent at the beginning of each new theme
Preschool and family	Activity afternoons	Included activities that were related to the ongoing theme and that parents could do together with their children; all activities were such that the children had practiced the things at preschool before the afternoon and could show their parents how to do the activities	Were organized at preschools by early educators; one activity for each EBRB theme
Family	Educational letter	General information on target behaviour of the theme: how the behaviour benefits health, increases skills and supports family life Tips for how to perform behaviour (e.g. increase PA as a family, increase fruit and vegetable consumption, decrease sugar intake, regulate children’s screen use) Information on the recommendations and normative information on the behaviours on average (e.g. average screen time or fruit and vegetable consumption of the study participants) Normative information on how other parents on average try to support the health behaviour in question	Were distributed to parents in paper through the preschool; one for each theme
	DAGIS e-mails	Included links to existing materials on the internet regarding the theme that was ongoing at that moment Two of the e-mails included video clips related to the theme	Were sent to families through the preschool; one for each theme
	Personal feedback	Personal feedback on child’s EBRBs based on the baseline measurements; alongside personal results, the averages of the study cohort were presented	Were sent directly to participants by researchers; one for each theme
	Map application	A map application where: Children and parents could tag their favourite places to be physically active and that they would recommend to other families Early educators could tag activity places where the group had visited during the preschool day and share the places with parents Early educators could create orienteering maps for themselves as well as for other groups	Was presented to parents by early educators during the screen-time-related theme
Family and child	Bingo board	Bingo board including fun activities that parents and children could try together at home	Were distributed to parents as a part of the educational letter
Child	MindUp™ curriculum	The curriculum aims to develop self-awareness, self-management, and self-regulation through attention awareness practices and lessons. Several activities include physical activity and eating (Maloney et al. 2016) (e.g. children were taught how the brain works by using fictive animal figures, and being mindful in their physical activity)	Was implemented in the preschool groups
	Activities related to EBRBs	Sensory-based activities and other activities related to fruit and vegetables Games and other activities (e.g. physically active Christmas calendar) to increase PA	Were implemented in the preschool groups
	Two storybooks	One of the storybooks was about the balance between inactivity and PA; the other storybook was about being brave with tasting food. At preschool, the stories were meant to be read with the whole group and adjusted for the group’s age; both stories included pictures, which made it possible to go through the stories using only the pictures	Were distributed to each preschool group as well as every family; family storybooks were attached to their educational letters

**Fig. 1:**
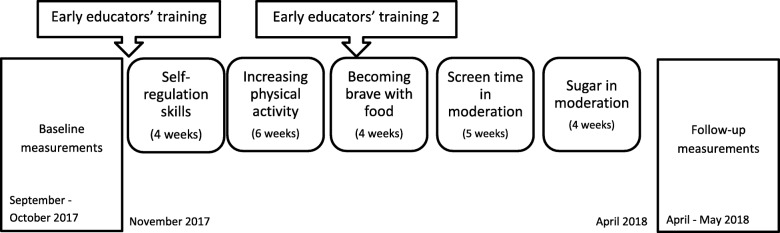
Time’ and themes of the DAGIS intervention programmes

The methods and the materials, such as encouraging low-budget family activities, were designed for the needs of low SES families. At this stage, we used knowledge from previous studies in the Nordic countries [57, 58], and we used the knowledge we had gathered through the focus groups in the autumn of 2014 [28, 29] and workshops in the autumn of 2016. We had workshops with the DAGIS steering group during 2016 and 2017, which yielded valuable knowledge about methods and materials appealing to low SES families. All these materials and methods were integrated with MindUp™ themes and into a schedule to form the DAGIS intervention programme (Table 3 and Fig. 1).

#### Feasibility testing and refinement of methods and materials

The interviews, conducted as a part of the feasibility testing, yielded knowledge about the early educators’ readiness for engaging with the materials and methods, their perceptions of the materials, how they had implemented the tested methods and materials, the interaction with parents about the materials sent home to families, and ideas that would promote the usability of the materials. Tested materials and methods were revised based on the feedback from parents and early educators during summer 2017. For example, the early educators suggested that the storybook should include a nursery rhyme in order to make the story more alive and easier to remember for the children. Another example was that early educators emphasized that it was important that they clearly knew the purpose and the aim of the materials and methods (such as materials, which will raise parents’ awareness or knowledge about EBRBs’, or methods which will engage children in PA etc), so that they could integrate and adapt the materials and methods in the best possible way. Revisions were made in the plans for the early educators’ training, and in the early educators’ manuals, so that the aims were explained more thoroughly. Members of the DAGIS research group visited a preschool in which MindUp™ had been applied for 2 years, and the preschool manager and early educators elaborated on their experiences in implementing the curriculum. They gave ideas about how to support the implementation. Early educators mentioned that more tangible activity ideas is needed when implementing the MindUp™ the curriculum, because they always have a lack of planning time. As a result, we gathered additional suitable activity ideas, methods, how to enhance self-regulations skills, and how to combine it with the promotion of EBRBs’.

### Results in step 5: planning adoption and implementation of the DAGIS intervention programme

The implementation plan for the DAGIS programme was developed in tandem with the programme design. The DAGIS steering group provided perspectives on how to engage the managers and early educators in the programme implementation and adoption. For example, they provided ideas about things and topics worth highlighting when communicating with managers and educators. The implementers for the DAGIS programme were preschool managers and early educators alongside the families (see Table 3). The implementation of the preschool component included three important methods: 1) appointing programme coordinators which were part of the research group, 2) organizing trainings for the early educators and managers in the intervention preschools, and 3) organizing a mentoring meeting for preschool managers and conducting mentoring visits in every intervention preschool group. To aid programme implementation, two coordinators supported preschool managers and early educators to implement programme activities at the preschools. Coordinators organized and carried out the early educators’ trainings as well as the mentoring visits. Further, the coordinators sent booster e-mails with additional activity ideas and tips to keep connected with preschool groups and boost the programme implementation.

Early educators’ trainings were organized at two different time points: before the programme start as two separate sessions lasting 6.5 h in total, and halfway through the programme as one training session lasting 3.5 h (see Fig. 1 and Table 3).

Teacher trainings have been used as one intervention strategy in many previous obesity-prevention/health-promotion interventions conducted in school or preschool settings [59–61]. Trainings have been shown to be important for the adoption of interventions and also suggested to be an essential element for successful interventions [46, 62]. The first training sessions covered the background and the aims of the programme, a knowledge-enhancing part that included EBRBs and how brains work and react and materials for the first three themes of the programme. The second part of the training covered a short repetition of the programme aims and background, perspectives from positive psychology and pedagogy, knowledge-enhancing EBRBs and materials for the last two themes of the programme. We chose to organize the trainings at two separate time points. We acknowledged that it was easier for the early educators to internalize the content and materials if it was split into two training sessions. Further, for example, Davis et al. [63] and Hoelscher et al. [64] have suggested that organizing trainings at several time points supports the implementation of programmes. Early educators’ manuals, including instructions and activity ideas for each theme, and supporting materials, were disseminated at the training sessions.

A meeting with all preschool managers was organized soon after the initial intervention trainings (Table 3). The aim was to hear preschool managers’ views on how early educators in their preschools accepted the programme and what kind of support could help them to get started with the programme. Additionally, reinforcing managers’ roles in supporting the programme implementation was on the meeting agenda, as managers’ role has been recognized as essential for successful implementation of programmes [65]. Coordinators visited each preschool group after 3–5 weeks from the programme start. These mentoring visits differed slightly between the groups according to the needs and wishes of the preschool. In some preschools, the coordinator visits were informal observation and discussion visits, while the early educators and children continued their normal tasks and routines in the preschool group. In some preschools, visits were organized as more formal meetings, having one person from each group participating in the meeting. The purpose of the meetings was to support early educators in the programme start.

The implementation of the programme at the family level included several methods (Table 3). The activity afternoons enhanced the interaction between the parents and between parents and the preschool personnel. Throughout the programme, families were reached through traditional educational letters produced in the DAGIS programme and distributed by the preschools to the families. Short e-mails about the programme themes were sent out through preschool e-mail lists. By using short videos in the e-mails, for example, the programme aimed to catch those parents who might have been less interested in reading educational letters. The implementation of the programme was also achieved by giving personal feedback on the children’s EBRBs based on results from the baseline measurements and by distributing a map application where families could mark and share with other intervention families their favourite places to be physically active outdoors. Additionally, the preschool personnel used the map application to share with families the outdoor places that the preschool group visited during preschool time.

### Results in step 6: evaluation of the DAGIS intervention

The evaluation of the DAGIS intervention was planned in tandem with the development of the DAGIS programme. The evaluation was planned to answer two main questions:

1. Did the conducted DAGIS programme have an effect on the primary and secondary outcomes in the DAGIS logic model of change and did the conducted DAGIS programme diminish SES differences in children’s EBRBs?
2. What were the processes in conducting the DAGIS programme like and how did the processes during the DAGIS intervention contribute to the project results?

In order to assess the effectiveness of the DAGIS programme, the evaluation included baseline and follow-up measurements, and it was planned to be conducted as a clustered randomized controlled trial (see Fig. 1). Preschools were planned to be randomized into intervention and control preschools after the baseline measurements. Power calculations showed that to be able to detect changes in children’s EBRBs in three SES groups, at least 432 children needed to participate in the DAGIS RCT study.

The effectiveness measurements were planned to be done both among adults and children. The baseline and follow-up measurements for adults included questionnaires for preschool managers, early educators and parents. Early educators evaluated self-regulation skills by using the Attention and Executive Function Rating Inventory, ATTEX instrument [66]. Parents reported children’s screen time at home by screen time diaries, and food consumption by electronic food frequency questionnaire (FFQ). Parent’s questionnaires included assessments of SES and the primary outcomes from the DAGIS logic model of change. Additionally, they evaluated children’s self-regulation skills by two instruments; the very short form Children’s Behavior Questionnaire [30] and a short ten-statement instrument [67]. Children wore accelerometers (Actigraph wGT3X-BT) during seven consecutive days in order to measure PA levels.

The recruitment plans for the DAGIS intervention, the DAGIS logic model of change together with the practical strategies, and the implementation plans, formed the basis for the process evaluation planning. For evaluating the intervention the RE-AIM (reach, effectiveness/efficacy, adoption, implementation, and maintenance) framework and the evaluation tools which are available at the RE-AIM website were utilized [68]. All five RE-AIM dimensions were to some extent included in the process evaluation plan (Table 4). A higher degree of implementation generally yields higher effects [69]. Therefore, in the process evaluation plan, a comprehensive assessment of the implementation degree was emphasized to broaden the understanding of possible intervention effects. Also the context and the readiness of the implementers were assessed. These factors have been shown to be crucial for successful implementations [23, 70]. Additionally, to the tasks delivered from the tools of the RE-AIM framework, the comprehensive process evaluation included detailed questions about following: dose delivered (e.g. was material delivered?), dose received (e.g. was activity conducted?), quality (e.g. was activity conducted as intended?), participant responsiveness (e.g. satisfaction), programme differentiation (e.g. what was essential?), readiness to implement the intervention (e.g. motivation, attitudes and self-efficacy), and the context (e.g. barriers and facilitators).

**Table 4:** The five RE-AIM (reach, efficacy/effectiveness, adoption, implementation, maintenance) dimensions and how these dimensions were planned to be measured through several components at different levels in the DAGIS intervention study

RE-AIM dimensions and the measured components	Measured at preschool level (yes/no)	Measured at parental level (yes/no)	Measured at child level (yes/no)
Reach			
Inclusion criteria	yes	yes	yes
Exclusion criteria	yes	yes	yes
Sample size	yes	yes	yes
Participation rate	yes	yes	yes
Characteristics of nonparticipants	yes	no	no
Efficacy/effectiveness			
Measures of outcomes for one follow-up	yes	yes	yes
Intention to treat analysis	yes	yes	no
Quality of life measure	no	no	no
Measure of robustness across subgroups (moderation analysis)	yes	yes	yes
Percent attrition	yes	yes	yes
Adoption			
Description of intervention location	yes	no	no
Description of staff who delivered intervention	yes	no	no
Level of expertise of delivery agent	yes	no	no
Adoption rate	yes	yes	no
Use of qualitative data to understand adoption	yes	no	no
Implementation			
Intervention type and intensity	yes	yes	no
Extent protocol delivered as intended	yes	yes	no
Consistency of implementation	yes	yes	no
Adoptions made to intervention during study	yes	no	no
Barriers for implementation	yes	yes	no
Use of qualitative data to understand implementation	yes	no	no
Maintenance			
Program components continued at 6 months following the completion of the intervention	yes	no	no
Characteristics of those continuing the program components	yes	no	no
Program modifications after 6 months of completion of the intervention	yes	no	no

The process evaluation targeted all essential stakeholders: preschool managers, early educators, parents, and children (Table 4). Preschools managers’ follow-up questionnaires had slightly different questions depending on belonging to the intervention or control group. Early educators also filled out follow-up questionnaires—separate questionnaires for control and intervention preschools. Early educators in intervention preschools filled out questionnaires after each programme training at the baseline and in the middle of the intervention. Early educators from each group filled in weekly logbooks assessing conducted relaxation moments, other conducted DAGIS intervention activities, and monthly activity afternoons arranged for parents. To get a deeper understanding of how managers and early educators perceived the DAGIS programme, interviews with all managers (*N* = 8) of intervention preschools and focus groups with early educators in six intervention preschools were conducted. The topics for the interviews were barriers and facilitators in implementing the DAGIS programme. The preschools chosen for the focus group were based on the motivation level for the programme at the baseline. The motivational level had been asked in a questionnaire at the first programme training for early educators. Two highly motivated, two middle-motivated, and two low-motivated preschools were chosen. The quantitative and qualitative measurements of barriers and facilitators for implementation enables triangulation in the evaluation process to get a deeper understanding of the adoption and implementation of the DAGIS programme. The parents’ questionnaire included process evaluation questions at follow-up. In addition, parents filled out an evaluation form at mid-intervention. Informal workshops were conducted with children in order to get a better understanding of children’s experiences and understanding of the programme.

## Discussion

The goal of this paper is to present the development process of the DAGIS intervention study aiming to decrease SES differences in children’s EBRBs and self-regulation skills by a preschool-based family-involving intervention and using the IM protocol [25] as a guiding tool for the development process. The DAGIS intervention study used the proportionate universalism approach, which meant that the intervention was delivered to the whole target group with a special emphasis on those needing it the most [15]. The result was the DAGIS intervention study, which was developed and conducted as a preschool clustered RCT between September 2017 and May 2018.

The IM protocol served as an easily applicable guiding tool in the planning process, and the good applicability has also been reported by others [71]. However, there are both pros and cons in using the IM framework, which in planning the DAGIS intervention study led to compromises at some steps. The use of the IM framework can to some degree make the planning process in the beginning, steps 1 and 2, longer and more burdensome than expected. The same remarks about extensive time demands were previously reported by others [71–73]. Because of the limited time resources, the researchers in the development of the DAGIS intervention study were forced to make some compromises. The change matrices for performance objectives, which are presented in step 2 in IM, were not formed as guided. This led to a limitation because the DAGIS logic model of change was not completed so that it could be presented as a proper model of change. Creating the change matrices would have enabled the DAGIS intervention to form more detailed performance objectives, which in turn would have improved the evaluation process. Still, the DAGIS intervention model includes behavioural outcomes at the child level as well as the adult level. The behavioural outcomes at the adult level are driven by DAGIS analyses from the comprehensive survey and an informal literature review. The planning of the effectiveness evaluation has been relying on the questions derived from the DAGIS survey. The DAGIS model also includes theory-driven personal determinants for adults’ behaviours, and the effectiveness evaluation has included questions regarding these determinants that are important for children’s EBRBs ( [41, 70], and unpublished results). Still, when evaluating the changing mechanisms for the effectiveness evaluation, a challenge might be that we did not create a matrix of change objectives. They point out the behaviour change that occurs when we want to change a determinant for the behaviour.

The developed DAGIS intervention study is, to the best of our knowledge, the first EBRB-promoting preschool intervention among children that applies the proportionate universalism approach [15]. It is a challenge to reach those who need it most, without stigmatizing any participants. One way to ensure that we had possibilities to reach children also from lower SES backgrounds, was to use the same method to invite municipalities to the intervention study, as was used in the survey: invite municipalities with as diverse a population as possible [74]. Having a whole municipality participating increased the possibilities of having children of all SES backgrounds. A Swedish research team applied the proportionated universalism by conducting a more intensive intervention in a deprived area than in other areas of Stockholm [75]. To apply the proportionate universalism approach in the DAGIS intervention study in that manner was challenging, due to that most preschools are mixed up with children from all kinds of SES backgrounds. Instead, we chose to develop a programme aimed to influence the most critical determinants for EBRBs in children with lower SES backgrounds. The determinants for children’s EBRBs, which were included in the DAGIS logic model of change, were derived from the multiple mediation analyses between family SES and children’s EBRBs. These were conducted in the first steps of the planning process (See chapter; Mediating factors between SES and children’s health behaviour). We included in the logic model of change only those determinants that in our analyses showed to be important mediators for associations between lower family SES and children’s EBRBs. By targeting those determinants in the DAGIS intervention, we aimed to change the critical determinants for lower SES family children, which will improve the lower SES children’s EBRBs. In addition, when we designed the programme methods and materials, they were designed to the needs of lower SES families, such as encouraging low-budget family activities, or producing easy to read materials.

The development of the DAGIS intervention applying the IM protocol has its limitations. As previously discussed, the limited time directed into not applying all tasks at each step. Some of the DAGIS intervention study researchers had either theoretical knowledge or practical experience of applying the IM framework, which could have strengthened the planning process [76]. The evaluation planning process was not entirely in tandem with the planning process of the DAGIS intervention. Part of the planning was still running when the DAGIS programme started. This can be seen both as a limitation and a strength. Soon after the first DAGIS training for the early educators, it emerged that deeper knowledge was needed about how the preschool managers perceived their role in the DAGIS intervention, and what the barriers and facilitators were for early educators to implement the programme. As the evaluation planning was still running, we were able to add qualitative evaluation methods to the process evaluation. Deeper knowledge about the context for an appropriate process evaluation is also highlighted in the MRC guidelines for process evaluation of complex interventions [23]. Conducting interviews with all intervention preschool managers in the middle of the programme, and focus group interviews with early educators after the follow-up measurements, will strengthen the evaluation of the DAGIS intervention study.

## Conclusion

To conclude, by applying the IM protocol in developing the DAGIS intervention study, a preschool-based family-involving programme was established. The development was time- and resource-consuming. Development started in 2014, and the intervention was conducted in September 2017 to May 2018. Applying the IM protocol had several advantages. The systematic planning, development, and running of the programme have reinforced a comprehensive evaluation that will enhance the knowledge of how to promote EBRBs and self-regulation skills among preschoolers and diminish SES differences in them.

## Supplementary information

**Additional file 1:** **Figure S1.** Theoretical underpinnings for adults as role models and actors for availability and accessibility in the DAGIS study. Theories adapted in the model: Social Cognitive Theory (Bandura [43]), Theory of Planned Behaviour (Ajzen [44]) and Self-determination Theory (Ryan and Deci 2000).

## Data Availability

Researchers interested in the data and materials from this study may contact principal investigator Eva Roos, eva.roos@folkhalsan.fi

## Abbreviations

DAGIS: Increased Health and Wellbeing in Preschools
EBRBs: Energy balance-related behaviours
IM: Intervention Mapping
PA: Physical Activity
RCT: Randomized Controlled Trial
SCT: Social Cognitive Theory
SDT: Self Determination Theory
SES: Socio-economic status
TPB: Theory of Planned Behaviour
