# Supplementary material for: Development of the DAGIS intervention study: a preschool-based family-involving study promoting preschoolers’ energy balance-related behaviours and self-regulation skills
Source: BMC Public Health. 2019 Dec 12;19:1670. doi: 10.1186/s12889-019-7864-0 (PMC6909522; doi:10.1186/s12889-019-7864-0)
Supplement: Supplementary file 1 — Additional file 1: Figure S1. Theoretical underpinnings for adults as role models and actors for availability and accessibility in the DAGIS study. Theories adapted in the model: Social Cognitive Theory (Bandura [43]), Theory of Planned Behaviour (Ajzen [44]) and Self-determination Theory (Ryan and Deci 2000). [file 12889_2019_7864_MOESM1_ESM.pptx]

## Slide 1
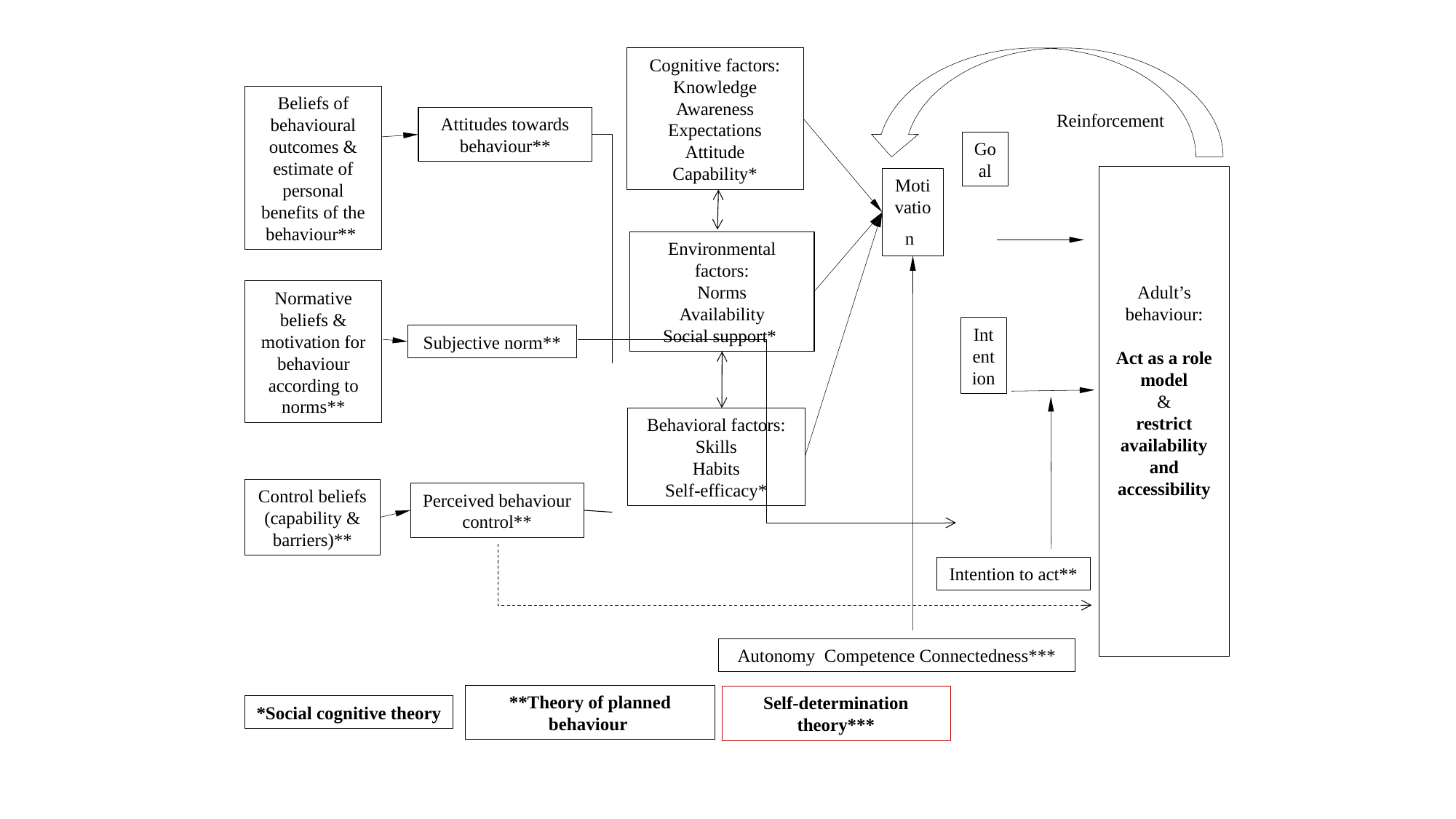

Cognitive factors:
Knowledge
Awareness
Expectations
Attitude
Capability*
Beliefs of behavioural outcomes & estimate of personal benefits of the behaviour**
Reinforcement
Attitudes towards behaviour**
Goal
Adult’s behaviour:
Act as a role model
&
restrict availability and accessibility
Motivation
Environmental factors:
Norms
Availability
Social support*
Normative beliefs &
motivation for behaviour according to norms**
Intention
Subjective norm**
Behavioral factors:
Skills
Habits
Self-efficacy*
Control beliefs (capability & barriers)**
Perceived behaviour control**
Intention to act**
Autonomy Competence Connectedness***
**Theory of planned behaviour
Self-determination theory***
*Social cognitive theory
